# Endoscopic Trans-Sphenoidal surgery; Efficacy and response in Pituitary Adenoma

**DOI:** 10.12669/pjms.342.14002

**Published:** 2018

**Authors:** Lal Rehman, Urooj Lal Rehman, Rukhshanda Jabeen, Raza Rizvi

**Affiliations:** 1 Dr. Lal Rehman, FCPS (NS), MS (NS). Department of Neurosurgery, Jinnah Post Graduate Medical Centre/ Jinnah Sindh Medical University, Karachi, Pakistan; 2 Dr. Urooj Lal Rehman, MCPS (Med), FCPS (Med), FCPS (Endocrinol). Department of Medicine, Endocrinology and Rheumatology, Jinnah Post Graduate Medical Centre/ Jinnah Sindh Medical University, Karachi, Pakistan; 3 Dr. Rukhshanda Jabeen, FCPS (Med). Department of Medicine, Endocrinology and Rheumatology, Jinnah Post Graduate Medical Centre/ Jinnah Sindh Medical University, Karachi, Pakistan; 4 Dr. Saleem Shah, FCPS PG Trainee (NS). Department of Neurosurgery, Jinnah Post Graduate Medical Centre/ Jinnah Sindh Medical University, Karachi, Pakistan

**Keywords:** Endoscopic Transsphenoidal Surgery, Pituitary Adenoma, Sellar Tumor

## Abstract

**Objectives:**

The purpose of the study was to access the efficacy and response of the endoscopic transsphenoidal surgery in pituitary adenomas.

**Methods:**

It was descriptive case study, conducted at Neurosurgery Department in collaboration with the Endocrine Unit (Medical Unit-II) Of Jinnah Post Graduate Medical Center Karachi from January 2015 to July 2017. Patients with sellar, supra sellar and para sellar tumors were enrolled in the study. Patients with prolactinoma and recurrent pituitary tumors were excluded. Data was analyzed using SPPS 17.

**Results:**

Sixty three patients were included in the study with mean age of 42±8.34 years. There were 40(63.5%) male patients and 23(36.5%) female patients with pituitary adenoma. Headache and visual impairment were the main presentation 55(87.3%) and 56 (88.8%) respectively. Out of all these patients the pituitary adenomas, 51(81%) patients had non secretory and 12 (19%) patients had secretory tumor. Out of these pituitary adenomas 53(84.1%) were macroadenomas and 10(15.9%) were microadenoma. Post operatively marked improvement in the headache was in all 100% patients and vision improved in 54 (96.4%). The most common post operative complication was cerebrospinal fluid (CSF) leak in 10 (15.9%) with 44 (69.8%) having no post complications at all. Mortality was reported to be just 1.6% i-e one patient.

**Conclusion:**

The endoscopic transsphenoidal approach for pituitary adenoma is the safest procedure with marked improvement in complications and reduction in patient’s hospital stay.

## INTRODUCTION

First line therapy for all the pituitary adenoma is surgical resection except for prolactinoma which has excellent response to pharmacological therapy.^1^ Endoscopic transsphenoidal surgery was developed under a minimally invasive surgical strategy. This endonasal transsphenoidal endoscopy eliminates needs of sublabial or trans-septal incision, use of any transsphenoidal retractor and nasal packing.^2^ The transsphenoidal approach for resection of a pituitary adenoma was first performed by Herman Schloffer more than 100 years ago.^3^ When compared to the techniques that utilize microscopes, endoscopic surgery permits a wider field of vision, better visualization of the supra and parasellar region and of the neurovascular structures (optic nerves, chiasm, carotid artery, and cavernous sinus).^4^ The introduction of the endoscope in the sella turcica brings to light the structures and the normal tissue/tumor interface thus facilitating the removal of tumor remains. The lateral field of vision is wider; the limitation on movement created by the use of the speculum is avoided.^5^

Several studies worldwide have demonstrated superiority of endoscopic approach to the microscopic approach.^4^,^5^ In this study we evaluated the advantages and limitation of endoscopic transsphenoidal surgery for pituitary adenoma in a public centre hospital which is one of the very few setups in Pakistan offering this procedure.

## METHODS

This is a descriptive study conducted in Neurosurgery Department in collaboration with Endocrine Unit of Jinnah Postgraduate Medical Centre, Karachi from January 2015 to July 2017. There were total 63 patients included in the study. Approval from hospital ethic committee was taken. Patients admitted for endoscopic transsphenoidal surgery with sellar or supra sellar tumors were included in this study. Patients who had prolactinoma or recurrent pituitary tumors were excluded.

For endocrine evaluation patients were referred to the endocrine unit of the hospital. They were examined from endocrine point of view, pre operatively and post operatively as well as at follow up. Serum plasma growth hormone (GH), insulin like growth factor- I (IGF-I), prolactin (PRL), adrenocorticotropin hormone (ACTH), morning cortisol, 24 hour urinary cortisol, thyroid stimulating hormone (TSH), leutinizing hormone (LH), follicle stimulating hormone (FSH), testosterone (T) and Estradiol (E2) levels were performed. Tumors were classified as secretary and non secretary on the basis of hormonal analysis.

Size and invasion of the adenoma was evaluated by magnetic resonance imaging (MRI) with and without contrast. Tumors were classified in two categorize on basis of the size: microadenoma (<1 cm); and macroadenomas (≥ 1cm). Pituitary CT scan was done to evaluate the nasal, sphenoid and sellar anatomy. Follow-up CT scan and MRI were done.

Under general anesthesia, patients were put in supine position with head flexed at 20 degree. Nasal packing was done with 4%xylocainsolusion mixed with adrenaline for 20 minutes. Endoscope was inserted in right nose and sphenoethmoidal recess was identified. Middle turbinate was considered as important land mark. After identification of sphenoidalostium, mucosa was stripped out and vomer with rostrum was resected. After entering into sphenoid sinus, mucosa was removed and sellar floor indetified. Drill was used to widen the exposure by drilling edges of the sphenoid sinus to identify carotid protuberences, planumsphenoidale and clival recess. Sellar floor was drilled and dura was exposed. Dura was opened to visualize the tumor. After central debulking, remaining tumor was sucked out. We used mainly 0 degree endoscope but 45 degree was used when needed to visualize the remaining tumor. After resection of the tumor, sellar reconstruction was done with bone pieces and fats taken from abdominal wall. In the 24 hours after the surgery, CT and endocrinological evaluations were conducted. Post operatively patients were observed for any complications like CSF leak, diabetes insipidus, hormonal disorders and visual problems. The data was evaluated by using SPSS 17; results were interrupted using frequency and percentages.

## RESULTS

A total of 63 patients presenting with pituitary adenoma were included in the study. There were 40(63.5%) male patients and 23(36.5%) female patients. The age ranged from 25 years to 60 years with mean age of 42 ± 8.34 years. Most common presenting complaints were headache in 55 (87.3%) and visual disturbance in 56 (88.3%) patient (Table-I). Pituitary adenomas were classified on the basis of size; 53(84.1%) were macroadenomas (≥1 cm) and 10(15.9%) were microadenomas (<1cm). Out of 53 macroadenoma 44 (69.85%) had supra sellar extension (Table-II). The pituitary adenomas were further evaluated for hormonal abnormalities; 51(81%) of adenomas were non secretary and 12(19%) were secretary adenoma which were mostly GH secreting 11 (17.5%). Due to the compression effect, 20 (31.7%) patient had hormonal deficiency.

**Table-I:** Presenting Complaints of Patients.

Presenting complaint		n 63 (100%)	
Headache		55 (87.3%)	
Visual problems	56 (88.8%)	Visual field defects	35 (62.5%)
		No Vision	2 (3.5%)
		Field defect with one blind eye	19 (33.6%)
Decreased libido		28 (44.4%)	
Acromegalic features		11(17.5%)	
Cushingoid features		1 (1.6%)	

**Table-II:** Classification of Pituitary Adenoma.

	Microadenoma	10 (15.9%)		
Size n 63	Macroadenoma	53 (84.1%)	Sellar	9 (16.9%)
			Sellar + Supra sellar	35 (66.0%)
			Sellar + Supra sellar + Parasellar	9 (16.9%)
Function n 63	Non Secretary	51(81%)
	Secretary	12(19%)	Acromegaly	11 (91.6%)
			Cushings disease	1 (8.3%)
	Pituitary deficiency because of mass effect	20 (31.7%)	Hypogonadism	10 (50%)
			Hypothyroidism	1 (5%)
			Pan hypopituitarism	9 (45%)

Gross total resection was possible in 41 (65.1%) (Table-III). After the procedure marked improvement in the presenting symptoms was observed especially headache improved in 100% of the patients. Levels of IGF 1 settled to normal in 50% of the acromegaly patients.

**Table-III:** Extent of Surgery, Outcomes; Improvement and Complications.

Extent of Surgery (n 65)			
Gross total resection	41(65.1%)	Microadenoma	10 (24.3%)
		Sellar	9 (21.9%)
		Sellar + Supra sellar	22(53.6%)
		Sellar + Supraseller + para sellar	0(0%)
Subtotal	14(22.2%)	Microadenoma	0 (0%)
		Sellar	0 (0%)
		Sellar +Supra Seller	9 (64.2%)
		Seller +Supra Seller+ Paraseller	5 (35.7%)
Partial	8(12.7%)	Microadenoma	0(0%)
		Sellar	0(0%)
		Sellar +Supra Seller	4(50%)
		Seller +Supra Seller+ Paraseller	4(50%)

*Outcomes (n 65)*		

Symptoms Improvement	Headache (n 55)		55 (100%)
	Vision (n 56)		54(96.4%)
Hormonal improvement	Acromegaly (n11 )		6 (54.5%)

*Complications (n=19, 30.1%)*		

CSF leaks			10(52.6%)
Temporary Diabetes Insipidus			3(15.7%)
Anterior Pituitary			3(15.7%)
Pan Hypopituitary (Ant. Pituitary +DI)			2(10.5%)
Death			1(5.2%)

There was no recurrence in patients with gross total resection. All the patients with subtotal resection and partial resection of the lesion had residual lesions. All residual tumors were given radiotherapy and medical treatment and follow up was done of all patients both in neurosurgery and endocrinology departments.

Post operative complications were seen in 19 (30.15%) patients. One (5.2%) patient expired because of meningitis. Most common complication seen in these patients was CSF leak in 10 (52.63%) patients (Table-III). Hormonal deficiency like temporary DI was seen in 3 (15.7%).

## DISCUSSION

Exclusive endoscopic transsphenoidal resection of pituitary adenomas when compared to the traditional microscopic approach is found to be safer and more efficacious.^6^ Advantages of this approach are; it provides a larger field of vision of the sella and surrounding structures, better understanding of anatomy, increased working field and reduced injuries to nasal cavity.^6^ Systematic reviews and international data have shown that the endoscopic approach is associated with a higher rate of gross total resection, decreased hospital stay and reduced observed post-operative complications.^7^ The Neurosurgery Department of Jinnah Post Graduate Medical Centre is one of the few centre’s in Pakistan performing this procedure. As no such published data was available from this region, the study was conducted to evaluate the outcomes of the endoscopic transsphenoidal pituitary surgery in a public centre.

In present study there was predominance of male patients as compare to female patients reported by the international data.^8^ The reason can be patients with prolactinomas were excluded from the study and prolactinomas are commoner in female rather than males.^9^ The age range for the pituitary adenomas in adult population reported worldwide is 12- 60 years.^3^,^4^ In this study the age range was 25-60 years. The reason for the difference in the minimum age of presentation in this study was firstly patients especially male patients did not recognize the symptoms like loss of libido earlier and secondly prolactinomas were excluded which is second most common type of pituitary adenoma presenting at earlier age especially in females as it causes irregular menstrual periods or amenorrhea.^10^

As with any space-occupying brain lesion, symptoms of elevated intracranial pressure may be elicited in patients of pituitary adenoma. Headache is reported to occur in 50% of macroadenoma patients; though migraine headache is most common, cluster headache and related headache subtypes are known to occur.^11^ In this study almost 87.3% patients presented with headache which was central and non radiating. Almost all (100%) of the patients after the surgery showed improvement in the headache.

Pituitary adenomas typically manifest as a result of mass effect on visual structures, endocrine abnormalities due to hormone hypersecretion or hyposecretion, or a combination of all.^11^ In this study visual impairement was the presenting complaint of 56 (88.3%) patients. The visual abnormalities varied from bitemporal hemanopia to complete loss of vision in one or both the eyes. Post operatively visual improvement was seen in 54 (96.4 %) patients this is comparable to the systematic review of the sixteen studies reporting higher rates of improved visual outcomes 91.1% in patients undergoing endoscopic transsphenoidal surgery.^12^ This is much higher and better than the 45.7% improved visual outcomes reported by the conventional open or transsphenoidal surgery.^12^

According to the size of the lesion, 53 (84.1%) of the pituitary adenomas were macroadenoma ≥ 1cm in size and 34 (64.1%) of these patients were male. This corresponds to findings from Nabarro who found that males were more likely to present with large pituitary tumors and women were more likely to present with small tumors.^13^ This is consistent with the observation that diagnosis of pituitary tumors may be delayed for males, giving the tumors a chance to grow larger before clinical detection.^14^

The rate of tumor removal depends upon its size and extension, which is a predicting factor for the outcome of surgery. With endoscopic endonasal approach, we can resect more tumor as compare to microscopic transsphenoidal and open surgery.^15^ In the present study, gross total resection of macroadenoma was possible in 41 (65.1%) patients comparable to 47.2% -96% reported by Fuyuwang et al.^6^

Pituitary adenomas are categorized as based on primary cell origin and type of hormone secreted. If the adenoma does not secrete a sufficient level of hormones to be detectable in the blood or to result in clinical manifestations, it is considered non secretory. Prolactinomas comprise 40% to 57% of all adenomas, followed by nonfunctioning adenomas (46% to 74%), growth hormone–secreting adenomas (11% to 13%), and adrenocorticotropic hormone (ACTH)–secreting adenomas (1% to 2%).^16^ As prolactinomas were not included in this patient series majority of patients had non secretory adenoma 52 (82.5%) presenting with symptoms of mass effect or hypopituitarism.

All 12 (19.0%) were secretary adenomas. The growth hormone secretion leading to acromegaly was seen in 11(91.66%). One (8.33%) patient had Cushing’s disease. Cure rate after endoscopic surgery was 50% in acromegalic patients.^17^ For secretory adenomas D’Haens et al.^18^ reported an increased cure rate (50%-65%) with endoscopic removal. Similarly cure rate is 57%, when pure endoscopic transsphenoidal approach is used for pituitary tumors, secreting growth harmone.^6^

Incidence of complications after endoscopic transsphenoidal surgery is 3.4 to 36.1% which are mostly CSF leak, dysfunction of the anterior lobe and diabetes insipidus.^19^ Over all complications observed in this study were 30.15% i.e. 19 patients. CSF leak was the commonest complaint in 10(55.5%) of these patients which did not require any surgical intervention CSF leakage could occur when cavernous sinus and diaphragmatic recesses are explored but mostly this resolves by itself. Post operative reversible diabetes insipid us appears to be either due to the surgical trauma or fall of diaphragm sella as the adenoma is removed resulting in stretching of pituitary stalk.^6^ Temporary DI was treated in 3 (16.6%) patients and they all improved.

The post operative anterior pituitary dysfunction results from excessive aspiration of the sella, inappropriate manipulation and removal of the normal gland along with the adenoma as well as damage due to the bipolar coagulation.^20^ In the macroadenoma, the rest of the pituitary land is compressed and thinned thus making it difficult to differentiate adenoma from the normal gland and may result in its damage.^21^ Post operative new anterior pituitary insufficiency affecting one axis or more than one axis was observed in 60% to 14% by Gondim et al. in a series of 50 patients^21^ which is comparable to 5 (27.7%) patients observed in this study. These patients were treated by endocrinologists with hormonal replacement therapy.

Mortality was observed in one (5.2%) patient and cause was meningitis a unique cause. Similar results have been observed by other studies in which minimal number of patients died 18.8% patients who had developed meningitis died.^22^ Hence mortality is not directly related to the surgical procedure.

## CONCLUSION

Endoscopic approach is not only minimally invasive but safe and efficacious, technique. It results in a higher gross total resection for both secretory and non secretory adenomas. Endoscopic transnasal surgery may lead to complications however they are of acceptable rate. In case of secretory adenomas, surgery alone is not sufficient to achieve remission and close coordination with endocrinologist is needed for control of hormonal dysfunction. For the better outcome of the pituitary adenoma the need of the time is advancement in endoscopy instruments, experienced neurosurgeons and endocrinologist, and an effective communication between the two as well as development and easy availability of the medications.

### Author`s Contribution

**LR:** Conceived, designed, statistical analysis, manuscript review.

**ULR:** Manuscript writing and data collection.

**RJ and SS** did data collection.
